# Age-associated SARS-CoV-2 breakthrough infection and changes in immune response in a mouse model

**DOI:** 10.1080/22221751.2022.2026741

**Published:** 2022-01-24

**Authors:** Yanxia Chen, Can Li, Feifei Liu, Zhanhong Ye, Wenchen Song, Andrew C. Y. Lee, Huiping Shuai, Lu Lu, Kelvin Kai-Wang To, Jasper Fuk-Woo Chan, Anna Jinxia Zhang, Hin Chu, Kwok-Yung Yuen

**Affiliations:** a State Key Laboratory of Emerging Infectious Diseases, Carol Yu Centre for Infection, Department of Microbiology, Li Ka Shing Faculty of Medicine, The University of Hong Kong, Pokfulam, Hong Kong Special Administrative Region, People’s Republic of China; b Department of Microbiology, Queen Mary Hospital, Pokfulam, Hong Kong Special Administrative Region, People’s Republic of China; c Centre for Virology, Vaccinology and Therapeutics, Hong Kong Science and Technology Park, Sha Tin, Hong Kong Special Administrative Region, People’s Republic of China; d Department of Clinical Microbiology and Infection Control, The University of Hong Kong-Shenzhen Hospital, Shenzhen, People’s Republic of China; e Academician Workstation of Hainan Province and Hainan Medical University-The University of Hong Kong Joint Laboratory of Tropical Infectious Diseases, Hainan Medical University, Haikou, People’s Republic of China

**Keywords:** Age, SARS-CoV-2, COVID-19, vaccination, re-infection, immune breakthrough

## Abstract

Older individuals are at higher risk of SARS-CoV-2 infection and severe outcomes, but the underlying mechanisms are incompletely understood. In addition, how age modulates SARS-CoV-2 re-infection and vaccine breakthrough infections remain largely unexplored. Here, we investigated age-associated SARS-CoV-2 pathogenesis, immune responses, and the occurrence of re-infection and vaccine breakthrough infection utilizing a wild-type C57BL/6N mouse model. We demonstrated that interferon and adaptive antibody response upon SARS-CoV-2 challenge are significantly impaired in aged mice compared to young mice, which results in more effective virus replications and severe disease manifestations in the respiratory tract. Aged mice also showed increased susceptibility to re-infection due to insufficient immune protection acquired during the primary infection. Importantly, two-dose COVID-19 mRNA vaccination conferred limited adaptive immune response among the aged mice, making them susceptible to SARS-CoV-2 infection. Collectively, our findings call for tailored and optimized treatments and prevention strategies against SARS-CoV-2 among older individuals.

## Introduction

The Coronavirus Disease 2019 (COVID-19) pandemic caused by severe acute respiratory syndrome coronavirus 2 (SARS-CoV-2) infection started in December 2019. As of December 2021, it has affected over 278 million people with over 5.4 million deaths [1]. This unprecedented pandemic has brought tremendous pressure to the global public health and medical service system. Although viral transmission and infection have been slowed down by vigorous infection control measures and massive vaccination programmes worldwide, the eradication of SARS-CoV-2 is unlikely.

SARS-CoV-2 is a new human-pathogenic betacoronavirus emerging from an animal reservoir [2]. SARS-CoV-2-infected patients may present with fever and respiratory symptoms, and most will recover in 1–2 weeks, while some progress to acute respiratory distress syndrome, multiple organ failure, and death [3–5]. Clinical reports indicated that the age of patients is an independent risk factor significantly associated with severe COVID-19 outcomes [6,7]. Recent reports suggested that patients over 65 are responsible for 80% of COVID-19 hospitalizations. Moreover, patients over 65 also suffer from a 20-fold greater COVID-19 fatality rate compared to those under age 65 [8]. Comorbidities, such as cardiovascular disease and diabetes mellitus in older adults, may contribute to severe outcomes, but the pathogenic mechanisms of severe COVID-19 in aged patients remain incompletely understood [9]. In addition to age-associated pathogenesis, how age modulates SARS-CoV-2 re-infection and vaccine breakthrough infection remains largely unexplored. In this study, we simultaneously compared aged mice and young mice on SARS-CoV-2 pathogenesis, re-infection, and vaccine breakthrough infections in a recently characterized physiological mouse model challenged by the wild-type SARS-CoV-2 B.1.1.7 variant [10]. We demonstrated that the interferon and adaptive antibody response were significantly impaired in aged mice compared with young mice upon SARS-CoV-2 challenge, leading to more severe disease manifestations. We further demonstrated that aged mice are more prone to re-infection and vaccine breakthrough infection despite two doses of mRNA vaccination. These features in aged mice were associated with a lower frequency of IgG-secreting cells and IFN-γ-secreting cells in vaccinated aged mice compared to vaccinated young mice. Overall, our study demonstrates that increased age results in more severe SARS-CoV-2 pathology, increased risk of re-infection, and higher risk of vaccine breakthrough infection. Our study suggests that treatment and prevention regimens should be tailored and validated for their effectiveness in older individuals.

## Materials and methods

### Viruses, cell lines, and biosafety

SARS-CoV-2 B.1.1.7 variant strain, isolated from a laboratory-confirmed COVID-19 patient in Hong Kong, was used in this study (EPI_ISL_1273444). The virus was cultured in Vero E6 cells, titrated for plaque-forming unit, and stored at −80°C before use. All experiments involving live SARS-CoV-2 were performed in the Biosafety Level-3 (BSL-3) facility of the University of Hong Kong (HKU) by following approved standard operating procedures.

### Animals

Female C57BL/6N mice were obtained from the Centre for Comparative Medicine Research of HKU, and kept in a BSL-2 animal laboratory with a 12-hour light–dark cycle, free access to water and diet. The mice were grouped as (1) young (6–8 weeks of age, average weight 20 gram ±2); (2) aged mice (52 weeks, 30 gram ±4). All the animal experimental procedures were approved by the Committee on the Use of Live Animals in Teaching and Research of HKU.

### SARS-CoV-2 B.1.1.7 infection of mice

10^3^PFUs of SARS-CoV-2 B.1.1.7 diluted in 20µl of phosphate buffered saline (PBS) were intranasally inoculated under anaesthesia by ketamine (100 mg/kg) and xylazine (10 mg/kg) [10,11]. As controls, mice were mock-infected with the same volume of PBS. Bodyweight of the infected animals was monitored for 14 days upon virus inoculation. Disease signs, including ruffled fur, hunched posture, lethargy, and laboured breathing, were observed and scored by giving one score to each signs. At 2-, 4- and 14-days post-infection (dpi), three to six animals in each group were euthanized to collect blood and tissues for virological, histopathological and immunological analyses.

## Vaccination procedure

A two-dose regimen of vaccination with COVID-19 mRNA Vaccine (BNT162b2, lot number 1B004A, BioNTech, Germany) at a 14-day interval was given by the intramuscular injection of 5 µg/per dose in 50 µl volume [12]. Control groups were injected with the same volume of normal saline. Blood samples were collected on day 14 and day 28 after the first dose of vaccination. Intranasal virus challenge with 10^3^PFUs of SARS-CoV-2 B.1.1.7 was performed 14 days after the second dose of vaccination. The mice were sacrificed at day 2 post-infection, and samples were harvested for virological and immunological analyses.

### Determination of viral gene copy and infectious viral titre in mouse tissues

The nasal turbinates (NT) and lung samples were homogenized and extracted for total RNA. SARS-CoV-2 RdRp gene copy number was determined by RT-qPCR with gene-specific primers. To determine the viral load, total RNA was extracted from 350 µl of clarified tissue homogenates using a MiniBEST Universal RNA extraction kit (Takara Bio Inc., Shiga, Japan). Real-time RT–PCR with primers for SARS-CoV-2 RNA-dependent RNA polymerase (RdRp) (Table 1) was performed on a LightCycler 96 system (Roche Applied Sciences, Indianapolis, USA) using a One-step RT–PCR reaction kit (Takara Bio Inc.) The expression of the house-keeping gene β-actin was determined in parallel for RNA normalization. [13]. To detect infectious virus in the tissues, NT or lung tissues taken at 2 and 4 dpi were homogenized in 1 mL of cold Dulbecco's Modified Eagle Medium (DMEM) supplemented with 1% penicillin and streptomycin. Tissue homogenates were clarified by centrifuge at 9000x g for 10 min at 4°C; supernatants were aliquoted and stored at −80°C until use. Infectious viral titre in homogenized tissue samples was determined by a 50% tissue culture infection (TCID_50_) assay in Vero E6 Cells [14]. The samples were 10-fold serially diluted and inoculated into Vero E6 monolayer in 96-well plates followed by incubation at 37°C for 1 h. The cells were further incubated for 72 h after washing away the inoculum with PBS. Cytopathic effect was examined, and 50% tissue infectious titres were calculated using the Reed & Munch endpoint calculation method as described previously.

**Table 1.** Sequences of primers and probes for real time RT-qPCR detection of viral load and mRNA gene expression of host cytokines/chemokines.

Gene name	Forward primer (5’ to 3’)	Reverse Primer (5’ to 3’)
*SARS-CoV-2 RdRp*	CGCATACAGTCTTRCAGGCT	GTGTGATGTTGAWATGACATGGTC
	Probe (5’ to 3’): FAM- TTAAGATGTGGTGCTTGCATACGTAGAC-lABkFQ	
*β-actin*	ATGGCCAGGTCATCACCATTG	CAGGAAGGAAGGCTGGAAAAG
	Probe (5’ to 3’): Cy5-AGCGGTTCCGTTGCCCTGAG-IABkFQ	
*IL-1β*	GCCTTGGGCCTCAAAGGAAAGAATC	GGAAGACACAGATTCCATGGTGAAG
*IL-6*	TGGAGTCACAGAAGGAGTGGCTAAG	TCTGACCACAGTGAGGAATGTCCAC
*TNF-α*	ATAGCTCCCAGAAAAGCAAGC	CACCCCGAAGTTCAGTAGACA
*IFN-α*	ARSYTGTSTGATGCARCAGGT	GGWACACAGTGATCCTGTGG
*IFN-γ*	AAGCGTCATTGAATCACACC	CGAATCAGCAGCGACTCCTT
*IP-10*	ATGACGGGCCAGTGAGAATG	GAGGCTCTCTGCTGTCCATC

### Histopathology, immunohistochemistry, and immunofluorescence study of mouse tissue sections

Formalin-fixed and paraffin-embedded lung and NT tissues were sectioned into 4 µm sections and stained with haematoxylin and eosin (H&E) for histopathological examination. To differentiate the severity of histopathology in the lung sections, histopathological changes, including pulmonary congestion, interstitial infiltration, alveolar infiltration and haemorrhage, were assessed and scored 0–4 as described previously [15,16]. Briefly, score 0 indicated normal histology of the lung section; score 1, only blood vessel congestion and peribronchiolar or perivascular infiltration were observed; score 2, in addition to 1, there is diffuse alveolar wall congestion and infiltration; score 3, localized alveolitis with air space infiltration, exudation or haemorrhage can be observed; score 4, diffuse alveolitis can be observed. Viral antigen expression in the tissues was stained with an in-house rabbit anti-SARS-CoV-2 nucleocapsid (N) antibody followed by FITC-conjugated goat anti-rabbit IgG secondary antibody (Thermo Fisher Scientific, Waltham, MA, USA) [11,17]. The slides were examined under the microscope. Images were captured using Olympus BX53 semi-motorized fluorescence or a bright-field microscope equipped with OLYMPUS CellSense Standard Software.

### Determination of cytokine and chemokine gene expression by qRT-PCR

Total RNA was extracted from clarified tissue homogenates and reverse-transcribed into cDNA with a PrimeScriptTM RT reagent kit (Takara Bio Inc.). Cytokine/chemokine gene expression levels were determined by qRT-PCR with gene-specific primers (Table 1) using the SYBR Premix Ex Taq II Kit (Takara Bio Inc.). The expression of the house-keeping gene, β-actin, was quantified in parallel for RNA normalization. The relative expression of cytokine/chemokine genes was analysed using the 2^−ΔΔCt^ method [18]. The expressions of cytokine/chemokine in mock-infected mouse tissues were used as baseline controls. Primer sequences are listed in Table 1.

### Microneutralization (MNT) assay

Serum samples were 2-fold serially diluted starting from 1:10 with PBS. Diluted serum was mixed with 100 TCID_50_ of SARS-CoV-2 and incubated at 37°C for 1 h. The virus/serum mixture was then inoculated to Vero E6 cells in a 96-well plate and cultured at 37°C for 72 h. Cytopathic effects (CPE) were observed. Neutralizing antibody titre was defined as the highest dilution of serum that completely inhibited the cytopathic effect.

### Fluorescent foci microneutralization (FFMN) assay

Two-fold serially diluted serum was mixed with 0.1 M.O.I. of SARS-CoV-2 virus and incubated at 37°C for one hour before inoculating into Vero E6 monolayer in chamber slides. After washing away the inoculum, the cells were incubated at 37°C for 6 h, and then fixed in cold acetone and methanol (1:1) for staining of SARS-CoV-2 N protein by immunofluorescence staining. The cells were examined under a fluorescent microscope, 400x magnification images were taken from 20 random microscopic fields. N protein-positive cells were counted using ImageJ. Percentage inhibition of virally infected cells by mouse serum was calculated against mock control serum-treated infection as previously described [19,20].

### Enzyme-linked immunosorbent assay (ELISA)

Inactivated and purified SARS-CoV-2 (2 µg/ml), purified recombinant SARS-CoV-2 nucleoprotein (N), or Spike protein receptor-binding domain (RBD) were coated into 96-well immunoplates (Nunc-Immuno Modules; Nunc A/S, Roskilde, Denmark) in 0.05M NaHCO_3_ (pH 9.6) and incubated for overnight at 4°C. To detect SARS-CoV-2 virus-specific antibodies in mouse sera, the plate was blocked with 1% bovine serum albumin at 37°C for 1 h; 2-fold serially diluted serum was added and incubated at 37°C for 1 h. The plate was then washed 6 times with PBS containing 0.05% Tween-20 and incubated with horseradish peroxidase (HRP)-conjugated secondary antibodies (Rabbit anti-mouse IgG, Goat anti-mouse IgG1, IgG2a, IgG2b, Abcam and Invitrogen) at 37°C for 1 h. After colour development with 3,3’,5,5’-tetramethylbenzidine solution (Life Technology) for 15 min at 37°C, the reaction was stopped with H_2_SO_4_. The optical density (OD) was read at 450 nm. The cut-off OD value was set at the mean OD of uninfected serum at all dilutions plus 3 standard deviations. The highest sample dilution, which produces an OD above this cut-off value, was taken as the antibody titre [21,22]. Albumin and haemoglobin concentrations were determined using a mouse albumin and haemoglobin ELISA kit (Abcam, Cambridge, UK) following the manufacturer’s instructions.

### Enzyme-linked immunospot (ELISPOT) assay

To detect virus-specific IgG secreting cells, 2.5 × 10^5^ cells/well single-cell suspension from mouse lung and spleen were seeded into ELISPOT plates coated with purified and inactivated SARS-CoV-2 virus (5µg/ml) for 48 h. IgG-producing cells were then detected by alkaline phosphatase (AP) conjugated-goat anti-mouse IgG antibody [19]. Virus-specific Interferon-γ secreting cells were determined by seeding 2.5 × 10^5^ cells/well single-cell suspension from mouse lung and spleen into mouse IFN-γ ELISPOT plates with the stimulation of SARS-CoV-2 RBD peptide pool and NP protein using the mouse IFN-γ ELISpot BASIC kit (Mabtech, Inc., Stockholm, Sweden) following the manufacturer’s instructions [23].

### Statistical analysis

All data were analysed with Prism 8.0 (GraphPad Software Inc). Student’s t-test, one-way or two-way ANOVA was used to determine significant differences among different groups. *P* < 0.05 was considered statistically significant.

## Results

### SARS-CoV-2 replicates more efficiently in the respiratory tract of aged mice than young mice upon virus exposure

**Figure 1.**
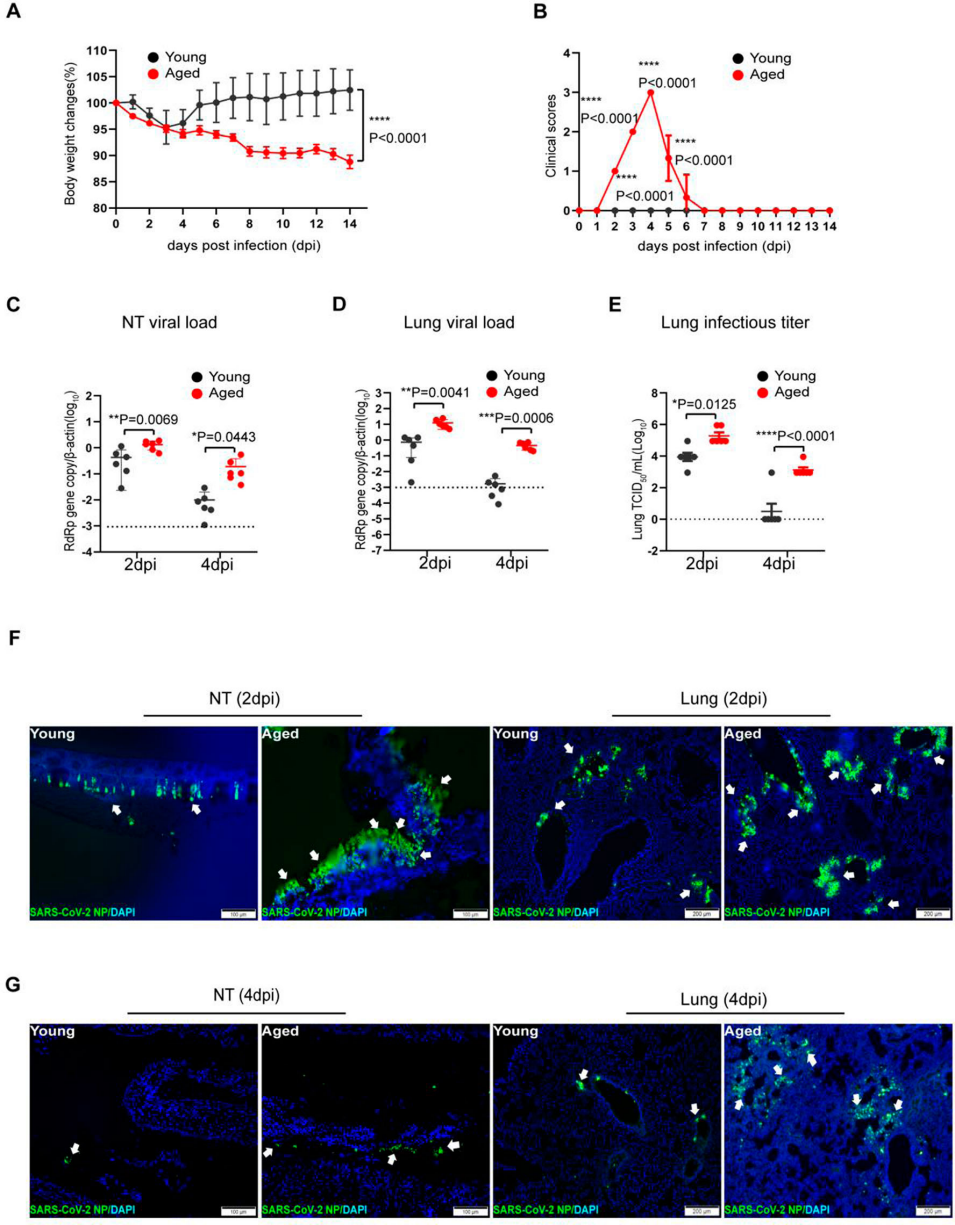
SARS-CoV-2 B.1.1.7 virus-infected wild-type C57BL/6N mice and replicated effectively in the upper and lower respiratory tissues of aged mice. Mice were grouped according to their age and inoculated with 10^3^PFUs of B.1.1.7 virus via the intranasal route. Body weight and signs of disease were monitored for 14 days after virus infection. (A) Body weight changes in young and aged mice. (B) Clinical scores of disease signs after virus infection. During daily monitoring of the infected mice, one score was given to each disease sign, including ruffled fur, hunched posture and laboured breathing. Highest total score = 3 per mouse. Data represent mean ± SD. *n* = 6 for each group. ****p < 0.0001 by Student’s *t*-test. (C and D) Real-time RT-PCR determined viral RdRp gene copies in the nasal turbinate (NT) (C) and lung tissues (D) of infected mice at 2 or 4 days post-virus infection (dpi). Data presented as copies of RdRp gene per copy of β-actin in log scale. Horizontal dashed lines indicate the detection limit of the assay. (E) Infectious virus titre in the lung tissues determined by 50% tissue culture infection dose (TCID_50_) assay on Vero E6 cells. Data represent mean ± SD. *n* = 6 for each group. *P < 0.05, **P < 0.01, ***P < 0.001, ****P < 0.0001 by two-way ANOVA. (F and G) Representative images of immunofluorescence staining of SARS-CoV-2 nucleocapsid protein (NP) in nasal turbinate (NT) and lung tissues of young and aged mice at 2 dpi (F), and 4 dpi (G). SARS-CoV-2 NP was stained green and indicated with white arrows. Cell nuclei were stained blue by 4’, 6-diamidino-2-phenylindole (DAPI). Scale bars = 100 µm.

We recently demonstrated that SARS-CoV-2 variants carrying the N501Y mutation in the spike protein infect wild-type C57BL/6N mice [10]. To explore age-related infection outcomes, we investigated the pathogenesis of SARS-CoV-2 in aged (52 weeks) and young (6–8 weeks) mice in parallel after being challenged by N501Y-bearing SARS-CoV-2 B.1.1.7 variant. Upon intranasal inoculation of 10^3^PFUs of B.1.1.7, the young mice transiently lost a maximum of 5% body weight from 2 to 4 days post-infection (dpi). In contrast, the aged mice lost significantly more weight (12%) without recovery at 14 dpi (Figure 1(A)). In addition, while infected young mice did not show any sign of disease, the aged mice showed ruffled fur, hunched postures, and laboured breathing, which were most severe at 4 dpi (Figure 1(B)). To compare the extent of virus replication in the upper and lower respiratory tissues of aged and young mice, we harvested nasal turbinate (NT) and lung from the infected mice at 2 and 4 dpi. Viral load assays showed that aged mice had significantly more RdRp gene copies in the NT (Figure 1(C)) and lung (Figure 1(D)) than the young mice. At 4 dpi, SARS-CoV-2 viral load was 19.3-folds (*p* = 0.0443) and 274.9-folds (*p* = 0.0006) higher in NT and lung of aged mice than young mice, respectively. Importantly, the aged mice also had significantly higher infectious virus titre in the lung compared to young mice at 2 dpi, while only one of the six young mice had a detectable infectious virus in the lung at 4 dpi, when the infectious virus titre in the lung of aged mice remained high (Figure 1(E)). Furthermore, by immunofluorescence staining of SARS-CoV-2 nucleocapsid (N) protein, we showed that, at 2 dpi, the abundance and tissue distribution of N antigen in NT were more intense in aged mice than young mice, which were localized to the nasal respiratory and olfactory epithelial cells [24] (Figure 1(F), left two panels). In the lung sections, N antigen was also more abundantly found in aged mice, spreading as multiple foci in the bronchiolar epithelium and adjacent alveoli. In contrast, N-expressing cells were less frequently found in the lung of young mice (Figure 1(F), right two panels). At 4 dpi, viral N protein expression in the NT and lung sections of young mice was substantially reduced compared to those in 2 dpi (Figure 1(G)). However, viral antigen was still readily detected in the NT and lungs as multiple foci in aged mice at 4 dpi (Figure 1(G)). These findings indicate that SARS-CoV-2 replicates more effectively in the respiratory tract of aged mice than young mice upon virus exposure.

### SARS-CoV-2 causes progressive inflammatory damage in the lungs of aged mice

**Figure 2.**
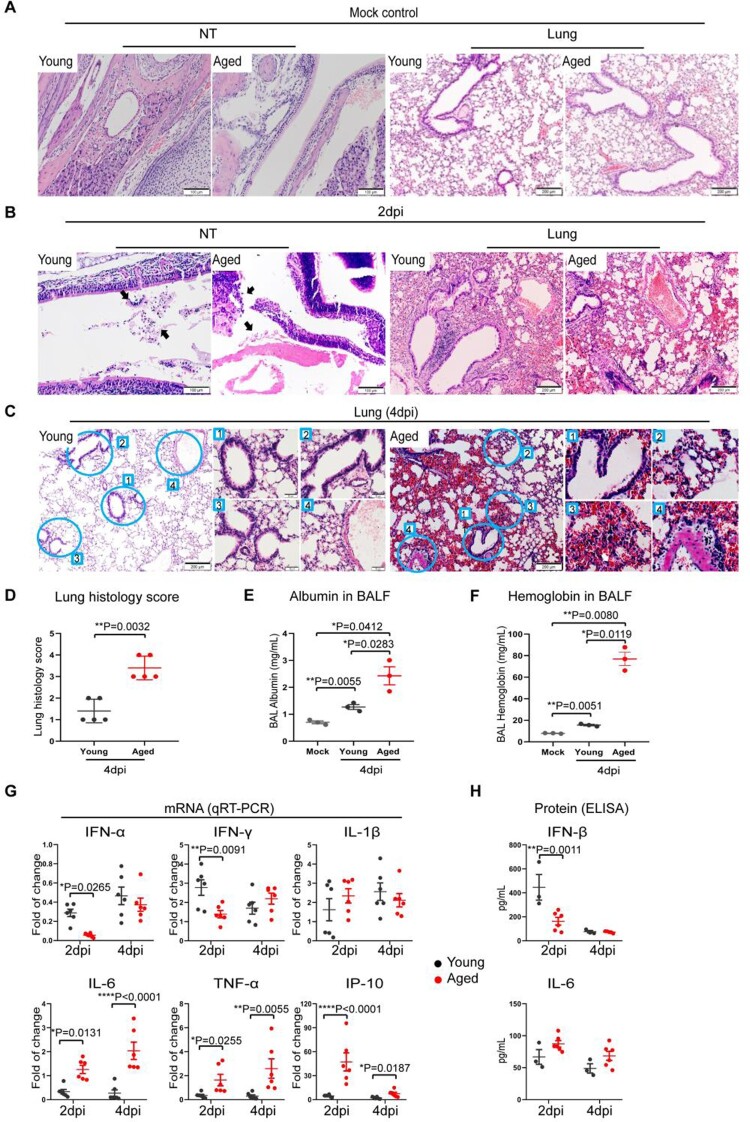
SARS-CoV-2 B.1.1.7 virus-infected and caused severe inflammatory damage to respiratory tissues in aged mice. A group of young and aged mice was inoculated with 10^3^PFUs of B.1.1.7 virus via the intranasal route. Tissue samples were collected and analysed at 2 and 4 days post-infection. Formalin-fixed and paraffin-embedded mouse nasal turbinate (NT) and lung tissue sections were stained by haematoxylin and eosin for histological examination. (A) H&E images showed NT and lung sections of mock infection controls of young and aged mice. (B) Representative images of NT section (left panels) and lung sections (right panels) of mice at 2 dpi. Solid arrows indicated nasal epithelium destructed and detached into the nasal cavity of mice. On the right shown representative H&E images of lung tissues. The lung of the young mouse showed peribronchiolar and perivascular immune cell infiltration and alveolar wall congestion. The lung of the aged mouse showed bronchiolar epithelium desquamation and endothelium infiltration in the blood vessel. (C) Representative H&E images lung sections at 4 dpi. The lower magnification images showed only mild alveolar wall thickening in the young mouse, while the lung of aged mice showed a large area of alveolar haemorrhage. The circled areas were magnified, showing (1) bronchiolar epithelial cell detachment and luminal cell debris, (2) alveolar space exudation, (3) alveolar space haemorrhage and (4) vasculature infiltration. All these features of inflammatory tissue damage were obvious in aged mice compared to young mice. Scale bars = 200 µm. (D) Scores for histopathological damage in the lung sections at 4 dpi. H&E stained mouse lung tissue sections were evaluated for the severity of bronchiolitis, alveolitis and vasculitis by the histopathologist. Data represent mean ± SD. *n* = 3–5 for each group. **P < 0.01 by student t-test. (E and F) ELISA assay determined the concentration of albumin (E) or haemoglobin (F) in the bronchiolar lavage fluid taken from infected mice taken at 4 dpi. Data represent mean ± SD. *n* = 3 for each group. *P < 0.05, **P < 0.01 by one-way ANOVA. (G) Inflammatory cytokine and chemokine in homogenized lung tissues of aged and young mice at 2 or 4 dpi. Relative mRNA expression levels of the cytokines were determined by qRT-PCR with gene-specific primers. House-keeping gene β-actin was included for the normalization of RNA concentration in each sample. (H) The protein concentrations for IFN-β and IL-6 were determined by ELISA. Data represent mean ± SD. *n* = 3–6 for each group. *P < 0.05, **P < 0.01, ***P < 0.001, ****P < 0.0001 by two-way ANOVA.

We next evaluated the histopathological changes of the infected aged and young mice (Figure 2(A–C)). Histological examination of the NT sections showed different degrees of submucosal immune cell infiltration and epithelium destruction at 2 dpi in young and aged mice (Figure 2(B)). The lungs of young mice showed localized interstitial inflammation as peribronchiolar and perivascular inflammatory infiltration and mild alveolar wall congestion and infiltration (Figure 2(B)), consistent with interstitial inflammatory changes. The tissue damages were more severe in the lungs of aged mice at 2 dpi manifested as alveolar capillary congestion, alveolar wall oedema, and localized alveolar haemorrhage (Figure 2(B)), which indicated the development of alveolitis after infection. Mock-infected mouse lung and NT were shown as control (Figure 2(A)). At 4 dpi, the lung of young mice demonstrated a mild degree of alveolar wall infiltration and blood vessel congestion, which were comparable or milder compared to those observed at 2 dpi (Figure 2(C), left panels). Unexpectedly, the lung of aged mice at 4 dpi further deteriorated compared to 2 dpi. The worsened histopathological damages were evidenced by pulmonary vasculitis, severe alveolitis, large areas alveolar haemorrhage, protein-rich exudation, and immune cell infiltration (Figure 2(C)). In keeping with these observations, our semi-quantitative lung histopathology scoring system revealed significantly higher scores in aged mice than young mice (Figure 2(D)). Furthermore, we detected a significantly higher concentration of albumin (Figure 2(E)) and haemoglobin (Figure 2(F)) in the bronchoalveolar lavage fluid harvested at 4 dpi in aged mice compared to young mice. Thus, our results indicate SARS-CoV-2 infection causes more severe inflammatory damage to air exchange structures in aged mice compared to young mice.

To understand how SARS-CoV-2 caused more severe diseases in aged mice, we evaluated the interferons and inflammatory cytokine/chemokine responses upon virus infection in the infected animal lungs. Our data revealed that the interferon response (IFN-α and IFN-γ) was more readily activated in young mice early in the infection (2 dpi). In particular, IFN-α and IFN-γ expression were 4.7-folds (*p* = 0.0265) and 2.0-folds (*p* = 0.0091) higher in the lung of young mice compared to aged mice, respectively, at 2 dpi (Figure 2(G)). In contrast, the expression of representative inflammatory mediators, including IL-6, TNF-α, and IP-10, was significantly higher in the lung of aged mice than those in young mice at 2 and 4 dpi (Figure 2(G)).

At the protein level, we detected a significantly higher level of IFN-β in the lung of young mice compared to aged mice at 2 dpi (446.1 vs. 181.6 pg/mL, *p* = 0.0011). Consistent with gene expression studies, we similarly detected an elevated level proinflammatory cytokine, IL-6, in the lung of aged mice compared to young mice at day 2 and day 4 post-infection (2dpi: 87.3 vs. 66.8 pg/mL, 4dpi: 73.0 vs. 49.0 pg/mL). Our data suggest the delayed interferon response and excessive inflammatory response in reaction to SARS-CoV-2 infection result in poor control on virus replication and exaggerated inflammatory damage in the lungs of aged mice.

### The adaptive antibody response against SARS-CoV-2 infection is impaired in aged mice

**Figure 3.**
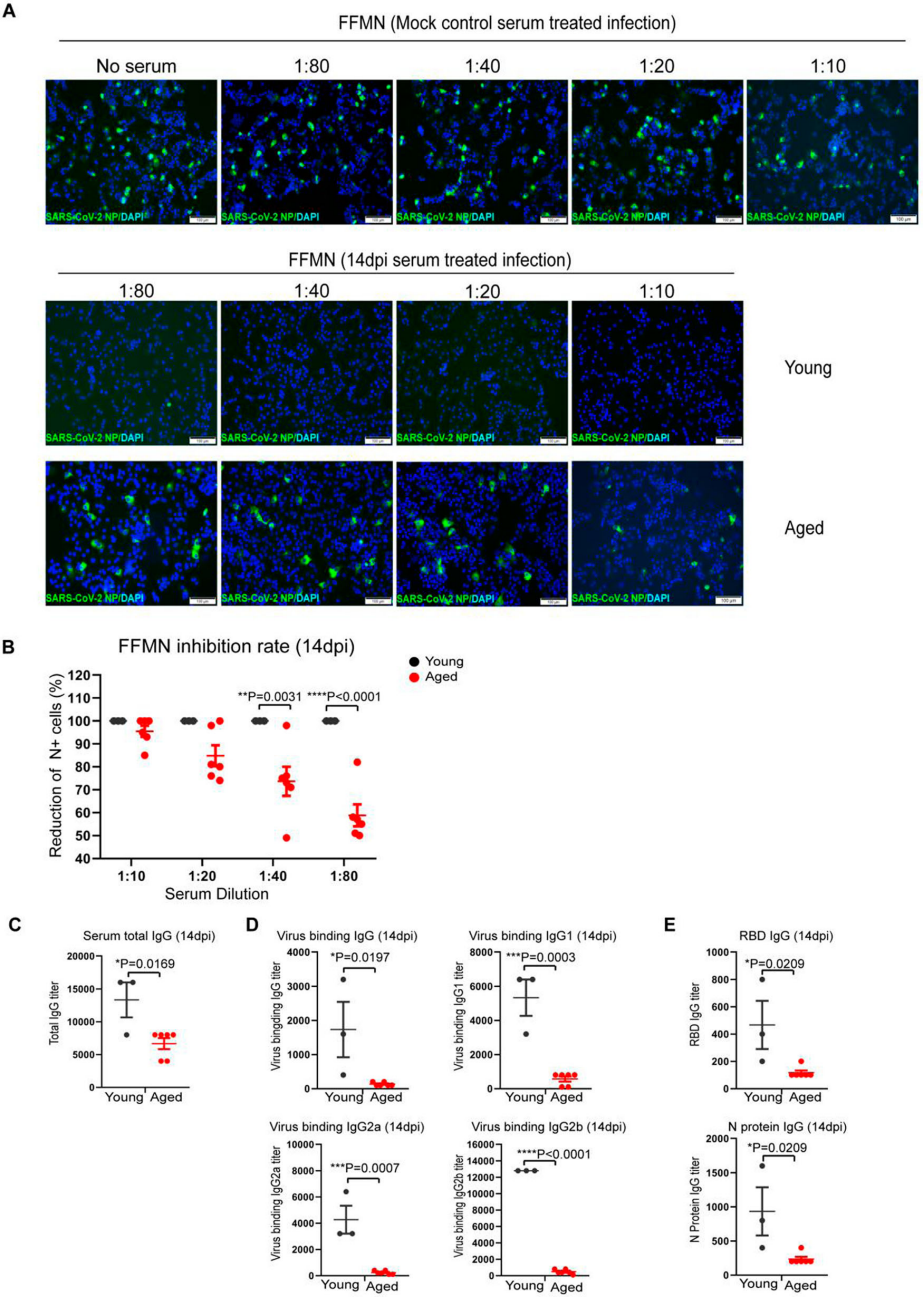
Serum antibody titres in SARS-CoV-2 B.1.1.7 infected aged and young mice determined by FFMN assay and ELISA. Fourteen days after intranasal inoculation of the B1.1.7 virus, the serum samples were taken for antibody determination, viral-neutralizing antibody determination on Vero E6 cells using fluorescence foci microneutralization assay (FFMN). Serum IgG and viral binding IgG were determined by ELISA with viral antigen-coated plates. (A**)** Representative images of immunofluorescence-stained SARS-CoV-2 NP in FFMN assay. SARS-CoV-2 B.1.1.7 virus (M.O.I. = 0.1) was allowed to react with the 2-fold serial diluted sera for one hour at 37°C before being added to Vero E6 cells. The cells were fixed and stained for SARS-CoV-2 NP after 6 h of incubation. Mock control mouse serum was tested parallel and shown in the top panel. The image in the middle panel showed no virus NP-positive cells in young mouse serum-treated infections, while the abundant NP-positive cells could be seen in aged mouse serum-treated infection (lower panel). (B) Percentage of reduction of N-positive cell after different mouse sera-treated infection versus mock controls in FFMN assay. (C–E) Mouse serum total IgG antibody (C), viral binding IgG and IgG subtypes (IgG1, IgG2a and IgG2b) (D) and IgG against RBD and N protein (E) in mouse serum determined by ELISA. Data represent mean ± SEM. *n* = 3–6 for each group. *P < 0.05, ***P < 0.001, ****P < 0.0001 by Student’s *t*-test.

To study the adaptive antibody responses after SARS-CoV-2 infection, the neutralizing activity of the serum antibody against live virus was determined by the fluorescence foci microneutralization (FFMN) assay, which assesses the ability of serum antibodies to block virus infection of host cells [20,25]. Our results showed that the serum (collected at 14 dpi) of young mice potently neutralized SARS-CoV-2, even at the highest dilution of 1:80 (Figure 3(A)). In contrast, the serum of aged mice demonstrated a substantially lowered capacity in neutralizing SARS-CoV-2 compared to that of young mice at all evaluated dilutions (Figure 3(A,B)). To further analyse the antibody response, we quantified serum IgG by ELISA. Our results showed that aged mice had significantly lower serum IgG than young mice. The total IgG and viral binding IgG, IgG1, IgG2a, and IgG2b subtypes in aged mice were all significantly lower than those of young mice by 2.0-folds (*p* = 0.0169), 13-folds (*p* = 0.0197), 9.4-folds (*p* = 0.0003), 18.2-folds (*p* = 0.0007), and 26.5-folds (*p* < 0.0001), respectively (Figure 3(C,D)). In agreement with these results, the IgG level against SARS-CoV-2 spike receptor-binding domain (RBD) and nucleocapsid (N) protein was significantly lower in aged mice than young mice by 4.0-folds (*p* = 0.0209) and 4.0-folds (*p* = 0.0209), respectively. Overall, these results indicate that the adaptive antibody responses are significantly impaired in aged mice.

### Prior SARS-CoV-2 infection insufficiently protects aged mice from re-infection

**Figure 4.**
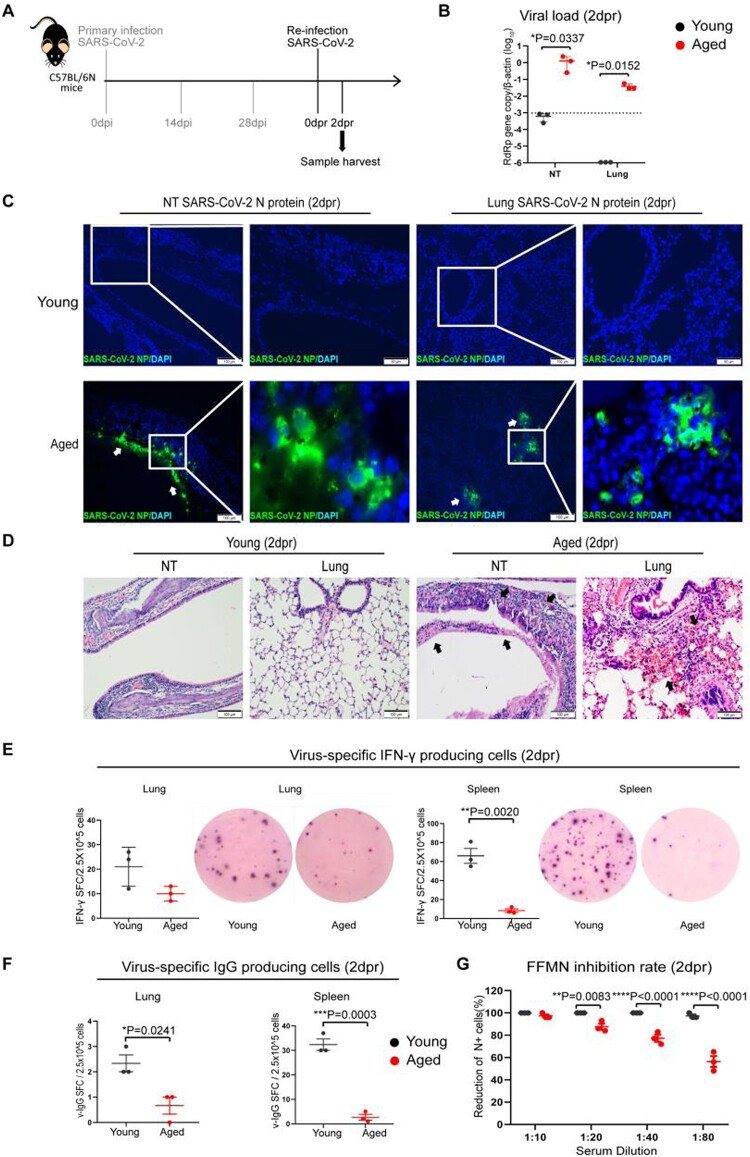
Viral load, tissue histological damages and immune responses after re-infection of mice with SARS-CoV B.1.1.7 virus. Mice recovered from 10^3^PFUs of B.1.1.7 infection were re-challenged with the same does of B.1.1.7 at 28 days after primary infection. Tissues were taken at 2 days post-re-infection (2 dpr) for virological, histological, and immunological analyses. (A) Schematic of infection and re-infection of mice. (B) qRT-PCR determined viral RdRp gene copies in the nasal turbinate and lung samples of re-infected mice at 2dpr. Data represent mean ± SD. *n* = 3 for each group. A horizontal dashed line indicates the detection limit of the assays. *P < 0.05 by two-way ANOVA. (C) Viral NP expression in the nasal turbinates and lung tissues of re-infected young (upper panel) and aged mice (lower panel). No NP-positive cells could be seen from NT and lung tissues of a re-infected young mouse. In contrast, immunofluorescence-stained SARS-CoV-2 NP was shown abundantly in the nasal epithelium and lung alveolar and bronchiolar epithelium of re-infected aged mice (white arrows). Squared areas were magnified. Scale bars = 100 µm. (D) Representative H&E images of nasal turbinate and lung sections of young mice and aged mice at 2 dpr showed no destruction of nasal epithelium and relatively normal alveolar histology with very mild pulmonary blood vessel congestion. The NT of aged mice showed submucosal immune cells infiltration and epithelium detached into the nasal cavity (black arrows). The lung showed diffuse alveolar haemorrhage and immune infiltration (black arrows)**.** Scale bars = 100 µm. (E) Interferon-γ-producing cell responses in re-infected mouse lungs and spleens collected at 2dpr. Viral-specific interferon-γ producing cells were detected by in vitro stimulation of single-cell suspension sample with SARS-CoV-2 RBD peptide pool and NP protein for 48 h and then visualized by staining with mouse IFN-γ ELISPOT kit. On right hand side is the representative images from the EISPOT assay. Data represent mean ± SD. *n* = 3 for each group. **p < 0.01 by Student’s *t*-test. (F) Viral-specific IgG-producing cells were detected by in vitro stimulation of lung or spleen single cells suspension with inactivated SARS-CoV-2 virus for 48 h. IgG-producing cells were visualized by staining with a mouse IgG ELISPOT kit. Data represent mean ± SD. *n* = 3 for each group. *p < 0.05, ***p < 0.001 by Student’s *t*-test. (G) Serum neutralizing antibody titre in the serum of mice at 2dpr was determined by FFMN assay. SARS-CoV-2 B1.1.7 virus (M.O.I. = 0.1) was allowed to react with the 2-fold serial diluted sera for one hour at 37°C before being added to Vero E6 cells. The cells were fixed and stained for SARS-CoV-2 N protein after 6 h of incubation. The percentage of reduction of NP-positive cells by serum treatment versus mock control serum was calculated. Data represent mean ± SEM. *n* = 3 for each group. ***p < 0.001, ****p < 0.0001 by Student’s *t*-test.

Next, we investigated how effective is the immunity acquired from previous SARS-CoV-2 infection against re-infection. We re-challenged aged and young mice with 10^3^PFUs of SARS-CoV-2 B.1.1.7 at 28 days post-primary infection and analysed the tissues at 2 days post-re-infection (2dpr) (Figure 4(A)). SARS-CoV-2 viral loads were largely reduced at NT and were completely undetectable from the lungs of young mice, suggesting convalescent young mice were protected from re-infection (Figure 4(B)). In contrast, SARS-CoV-2 viral load was readily detected in NT and the lungs of aged mice upon re-infection. Notably, while SARS-CoV-2 replicated to a lower level in the lung of aged mice upon re-infection compared to the primary infection, with relative viral RdRp copy measured as 12.44/β-actin in the primary infection versus 0.44/β-actin in re-infected mouse lungs (*p* = 0.0309) (Figure 1(D) and 4(B)), Viral load in the NT of re-infected aged mice reached a similar level as those of the primary infection at 2 dpi, 1.32 vs. 1.27 RdRp copy/β-actin (Figure 1(C) and 4(B)). Besides the presence of SARS-CoV-2 N antigen by immunofluorescent antigen staining in NT of aged mice, multiple foci of N protein were detected in the bronchiolar epithelium and adjacent alveoli in the lung of re-infected aged mice. In stark contrast, N antigen was not detectable in all NT and lung sections of young mice after re-infection (Figure 4(C)). Histological examinations revealed epithelial tissue destruction in the NT and peribronchiolar/perivascular infiltration, alveolar capillary congestion, and localized alveolar haemorrhage in the lung tissues of aged mice upon re-infection (Figure 4(D)). Furthermore, we demonstrated that the number of viral-specific interferon-γ-producing cells and viral-specific IgG-producing cells were substantially lower in the lung and spleen of aged mice at 2 days after re-infection than those of young mice (Figure 4(E,F)). Serum neutralizing antibody titre was significantly lower in aged mice than young mice upon re-infection (Figure 4(G)). Taken together, these findings indicate that the convalescent aged mice remain susceptible to re-infection, which is largely due to the impaired adaptive immune responses.

### Vaccination-induced immune responses incompletely protect aged mice from SARS-CoV-2 infection

**Figure 5.**
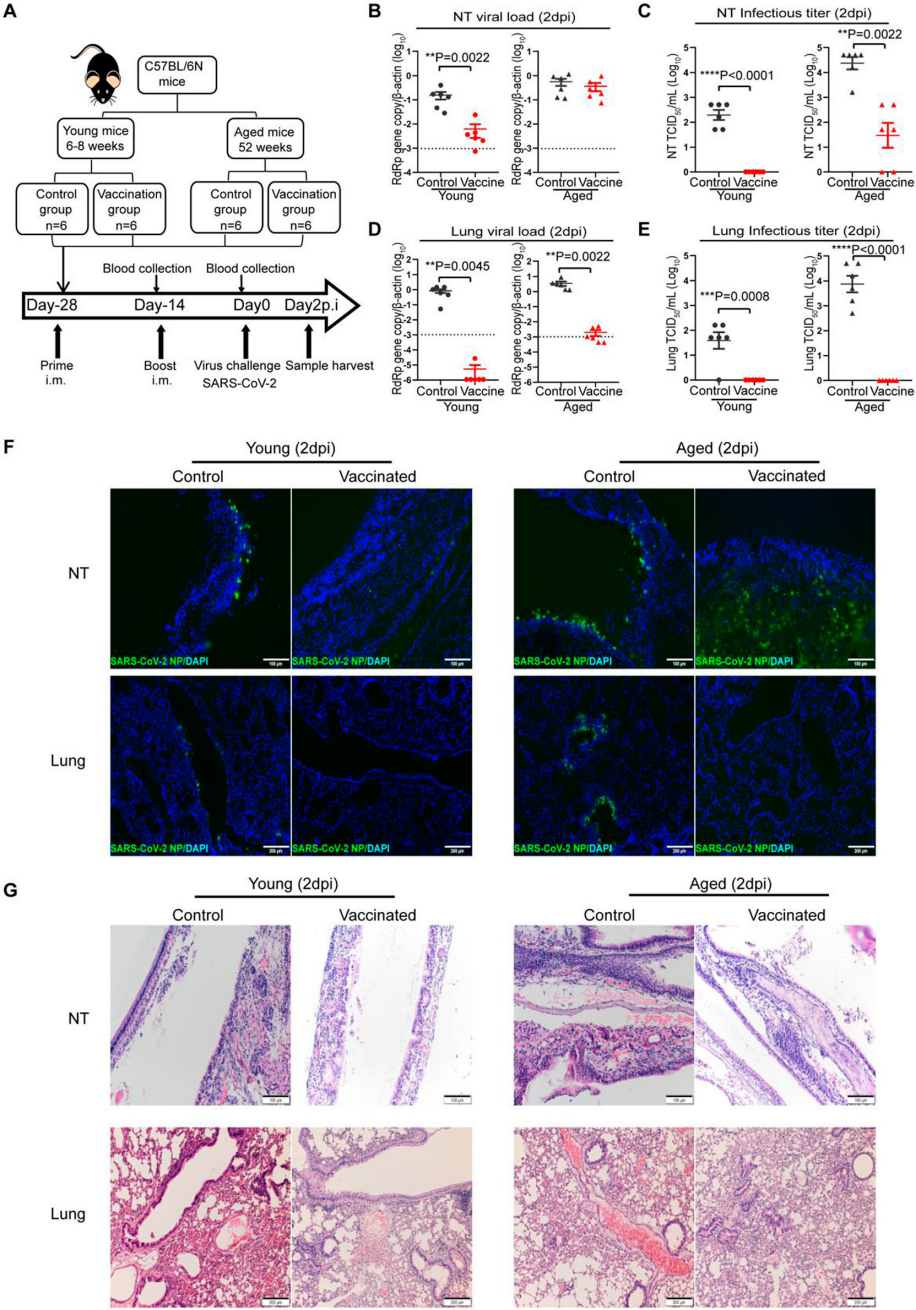
Viral load, histopathological changes in vaccinated mice challenged SARS-CoV-2 B.1.1.7 virus. (A) Schema of immunization of aged and young mice through intramuscular injection of COVID-19 mRNA vaccine and virus challenge of vaccinated mice. Young and aged mice were given two doses (5 µg of antigen per mouse) intramuscular injection COVID-19 mRNA vaccine at a 14-day interval, normal saline (NS) as control. Serum samples were taken 14 days after the first injection and again 14 days after the second dose injection. The mice were challenged with 10^3^PFUs SARS-CoV-2 B.1.1.7 virus 14 days after the second vaccination. Blood and tissue samples were taken on day 2 post virus challenge (2 dpi) for immunology, virological, and histopathological analyses. (B–E) Real-time RT-PCR determined viral RdRp gene copies (B) and infectious virus titre (C) determined by TCID_50_ assay on Vero E6 cells in the nasal turbinate tissues of infected mice at day 2 post-virus infection. Real-time RT-PCR determined viral RdRp gene copies (D) and infectious virus titre (E) determined by TCID_50_ assay on Vero E6 cells in the lung tissues of infected mice at day 2 post-virus infection. Data presented as copies of RdRp gene per copy of β-actin in log scale. Data represent mean ± SD. *n* = 6 for each group. A horizontal dashed line indicates the detection limit of the assays. **P < 0.01, ***P < 0.001, ****P < 0.0001 by Student’s t-test. (F) Representative images of immunofluorescence of viral N protein in the nasal turbinates and lung tissues of vaccinated or NS control young and aged mice at 2 dpi after virus challenge. (G) Representative H&E images of the nasal turbinated and lung tissues of vaccinated or NS control young and aged mice at day 2 post-infection.

Next, to evaluate how age will affect the outcome of COVID-19 vaccination, we immunized aged mice and young mice with a two-dose intramuscular mRNA vaccination regimen illustrated in Figure 5(A). The vaccinated mice were challenged by 10^3^PFU of SARS-CoV-2 14 days after the second vaccination dose. NT and lung tissues were harvested and analysed at 2 days post virus challenge. In the NT, our results showed that the mRNA vaccination significantly reduced SARS-CoV-2 viral load in young mice by 25.4-folds (*p* = 0.0022) compared to unvaccinated controls (Figure 5(B)), and completely abolished the infectious virus production (Figure 5(C)). Strikingly, SARS-CoV-2 viral load was detected at largely the same level in the NT tissues of vaccinated aged mice compared to the unvaccinated control aged mice (Figure 5(B)). In addition, SARS-CoV-2 infectious titres were readily retrieved from the NT of 4/6 vaccinated aged mice (Figure 5(C)). In the lungs, SARS-CoV-2 viral load was largely reduced to undetectable levels in vaccinated young mice. In comparison, SARS-CoV-2 viral load was still readily detectable in the lung of vaccinated aged mice though at a significantly lower level than unvaccinated controls (Figure 5(D)). Nevertheless, no infectious titre was retrieved from the lung tissues of either vaccinated young or aged mice (Figure 5(E)). Viral N protein was not detected from the NT and lung of vaccinated young mice and the lung of vaccinated aged mice by immunofluorescence staining. In contrast, we did not detect a decrease in viral N protein expression in the NT of vaccinated aged mice. These results indicate mRNA vaccine induces potent protection in the lower respiratory tract in young and aged mice, but is less effective in preventing virus infection and replication in the nasal cavity, particularly in the aged mice (Figure 5(F)). In keeping with the virological assessments, histopathological examinations showed that pulmonary inflammation was substantially ameliorated in vaccinated young mice, but to a lesser degree in vaccinated aged mice. Severe virus-induced epithelium destruction and submucosal immune cell infiltration were frequently detected in the NT of vaccinated aged mice (Figure 5(G)). These results indicate that mRNA vaccination insufficiently protects the upper respiratory tract in aged mice, which may allow SARS-CoV-2 vaccine breakthrough infections.

**Figure 6.**
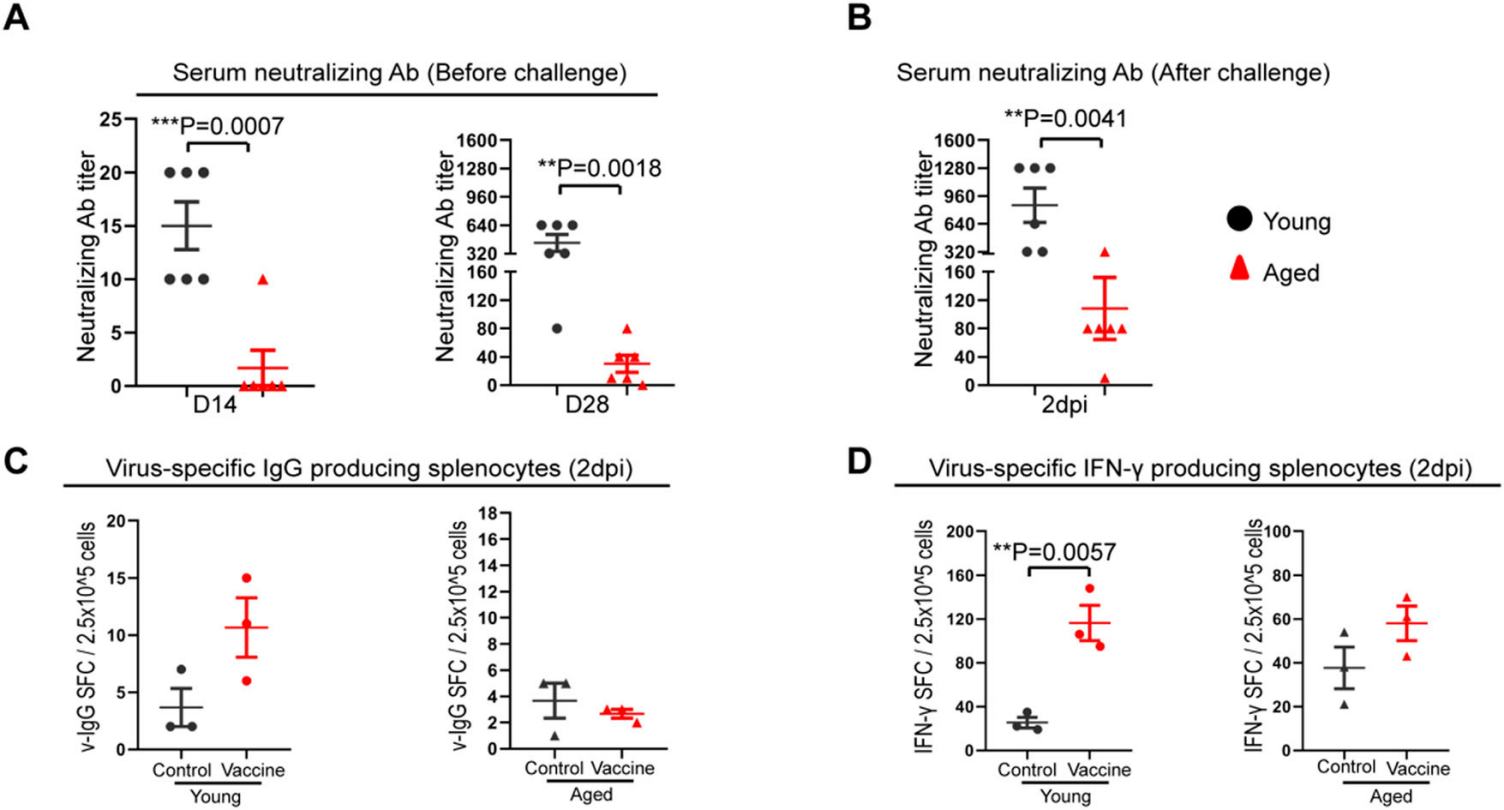
Immune responses after COVID-19mRNA vaccination and virus challenge in aged and young mice. (A) Vaccination-induced serum neutralizing antibody titre against SARS-CoV-2 B.1.1.7 at day 14, 28 after the first dose of vaccine. (B) Serum-neutralizing antibody titre against SARS-CoV-2 B.1.1.7 at day 2 post-virus challenge. Data represent mean ± SEM. *n* = 6 for each group. **P < 0.01 by Student’s *t*-test. (C) Viral-specific IgG-producing cells were detected by in vitro stimulation of spleen single cells suspension with inactivated SARS-CoV-2 virus for 48 h. IgG-producing cells were visualized by staining with a mouse IgG ELISPOT kit. (D) Interferon-γ-producing cell responses in the spleens collected at 2 dpi from vaccinated mice. Viral-specific interferon-γ producing cells were detected by in vitro stimulation of single-cell suspension sample with SARS-CoV-2 RBD peptide pool and NP protein for 48 h and then visualized by staining with a mouse IFN-γ ELISPOT kit. Data represent mean ± SD. *n* = 3 for each group. **P < 0.01 by Student’s *t*-test.

To further analyse the immune response induced by mRNA vaccination, we quantified the neutralizing antibody titre at different times after vaccination. At 14 days after the first vaccination dose, low level of serum neutralizing antibody responses were detected in young mice [geometric mean titre (GMT) = 14.1], which was still significantly higher than that of aged mice among which only 1/6 aged mice had a titre of 1:10 and 5/6 had undetectable titre (*p* = 0.0007) (Figure 6(A)). Consistently, the neutralizing titre was dramatically augmented by boost vaccination in the young mice (GMT = 359.1), while the GMT of aged mice increased to 26.3, which was significantly lower than that of young mice (*p* = 0.0018) (Figure 6(A)). Next, we determined the immune memory responses upon virus challenge and showed that the serum neutralizing antibody titre in vaccinated aged mice was approximately 10-folds lower than that of young mice (GMT = 71.2 in aged mice vs. GMT = 718.3 in young mice, *p* = 0.0041) at day 2 after SARS-CoV-2 challenge (Figure 6(B)). Moreover, we examined the frequency of virus-specific IgG-secreting cells and virus-specific IFN-γ-secreting cells in the spleen at 2 days post virus challenge. Virus-specific IgG-secreting cells and virus-specific IFN-γ-secreting cells in vaccinated aged mice were found substantially lower than vaccinated young mice and were similar to unvaccinated aged mice controls (Figure 6(C,D)). These findings suggested that the activation of vaccine-induced immune memory upon virus challenge is substantially weakened in aged mice.

Comparing adaptive immune responses elicited by vaccination with that by virus infection, vaccination-induced 1.8-fold more IFN-γ-producing splenocytes and 3-fold fewer IgG producing-splenocytes in young mice. Similarly, IFN-γ-producing splenocytes were 7-folds more abundant in vaccinated aged mice than in re-infected aged mice. However, the frequency of IgG-producing splenocytes in vaccinated aged mice was as low as that in the re-infected aged mice (Table 2). These findings suggest that although B cell and T cell immune responses are impaired in aged mice compared to young mice, mRNA vaccination still confers better T cell responses than natural virus infection in aged mice.

**Table 2.** Comparison of vaccination and infection-induced IgG or IFN-γ secreting splenocytes.

		Vaccination^a^	Re-infection^b^	*P* value
Young mice (*n* = 3)	IgG (SFC^c^/2.5 × 10^5cells)	10.7	32.3	0.0034
	IFN-γ (SFC/2.5 × 10^5cells)	116.3	66	0.0484
Aged mice (*n* = 3)	IgG (SFC/2.5 × 10^5cells)	2.7	2.7	Ns
	IFN-γ (SFC/2.5 × 10^5cells)	58	8.3	0.0037

^a^ 2 days post-infection of vaccinated mice.

^b^ 2 days post-re-infection.

^c^ SFC, Spot Forming Cells.

## Discussion

SARS-CoV-2 infection results in an overall mortality rate of approximately 2%. However, the risk of SARS-CoV-2 infection grows proportionally with age, and older individuals are at disproportionately higher risk of developing severe COVID-19. In particular, patients over 65 are responsible for 80% of COVID-19 hospitalizations and suffer from a 20-fold higher COVID-19 fatality rate compared to those under 65 years old [8]. To understand SARS-CoV-2 pathogenisis and vaccination responses, rodent models have been extensively used because of their wide availability and short life span. These small animal models have also been used to study human aging and age-related diseases. It has been shown that C57BL/6N mice start to have age-related conditions such as cardiovascular pathologies, metabolic disorders and increased basal expression levels of inflammatory cytokines from 10 months of age [26]. A study by Pinchuk L.M. et al. compared the age-related changes of the immune system in C57BL/6N mice between 10-month and 18-month age groups, which demonstrated their similar levels of CD4, CD8 T cells and antigen presentation cells, while the CD19 positive cells in PBMC and spleen increased with age [27]. In this study, we used C57BL/6N mice at 6–8 weeks as young mice and 12 months as aged mice to investigate age-associated SARS-CoV-2 pathogenesis, re-infection, and vaccine breakthrough infections using our recently characterized wild-type mice infection model [10]. First, we demonstrated that innate interferon response and adaptive antibody response against SARS-CoV-2 infection are significantly impaired in aged mice compared to young mice. These immune changes resulted in more efficient virus replication in the upper and lower respiratory tissue, excessive inflammatory response, and more severe histopathological damage to air-exchange structures in aged mice, consistent with the findings from other animal models [28,29]. Second, we demonstrated that the aged mice were more prone to re-infection. In particular, SARS-CoV-2 replication in the NT of aged mice upon re-infection was essentially the same as the primary infection in terms of viral load and viral antigen expression. Third, after two doses of COVID-19 mRNA vaccination, abundant infectious virus titre was still readily retrieved from the NT of aged mice upon virus challenge, indicating that the mRNA vaccination-induced immune responses incompletely protected aged mice from SARS-CoV-2 infection. Overall, our study demonstrated that age is a key determinant of SARS-CoV-2 pathogenesis and that ageing increases the risk of SARS-CoV-2 re-infection and vaccine breakthrough infections.

Emerging SARS-CoV-2 variants, including B.1.1.7 with an N501Y mutation in its spike protein that increases their binding to the mouse ACE2, allowing it to infect mice and rats as reported previously [10]. In this study, we further expanded this mouse model for age-related infection and infection prevention studies. The combined use of the natural N501Y-carrying SARS-CoV-2 variants and wild-type mice represent a new model for SARS-CoV-2 research with many strengths. First, compared with the hACE2-transgenic mice model with aberrant hACE2 expression [30], the endogenous ACE2 expression in wild-type mice is more physiologically relevant. Second, the N501Y-carrying SARS-CoV-2 variants used for this model are natural and do not carry laboratory-acquired changes generated from serial passages in mice [31,32]. Third, infection with N501Y-carrying SARS-CoV-2 variants is compatible with all existing knock-out and knock-in mouse models that will greatly facilitate further functional studies on host genes and pathways. Using this mouse model, we demonstrated that aged mice suffer from severe diseases upon SARS-CoV-2 infection, which could be attributed to the impaired interferon and adaptive immune response and more effective virus replication in respiratory tissue that caused immunopathological damages.

Re-infection of SARS-CoV-2 has been well-documented by us and others [33,34], but whether older individuals are more prone to SARS-CoV-2 re-infection remains incompletely understood. We found an increased incidence of re-infection in aged mice compared to young mice, in particular, a similar level of SARS-CoV-2 replication in the NT upon re-infection compared to that of the primary infection [primary infection vs. re-infection in NT at 2dpi: 1.31955 vs. 1.26542 RdRp copy/β-actin (log10)], which suggested lower protective effects to the upper respiratory tissue were conferred by the primary infection. To explain this high-frequency of re-infection, we found that at 14 days after the primary SARS-CoV-2 infection, serum levels of total IgG, virus binding IgG, IgG1, IgG2a, IgG2b were all significantly lower in aged mice than young mice. This indicated the Th1 and Th2 antibody responses to SARS-CoV-2 were impaired in aged mice. Subsequently, neutralizing activity in the serum of aged mice was so low that it could only be detected by the FFMN assay [20,25], while not detectable with the standard microneutralization assay. Importantly, re-infection in the current study has utilized the same SARS-CoV-2 variant strain as in the primary infection, while in the real-life scenario, the re-infection is more likely due to a SARS-CoV-2 variant different from that of the primary infection. Thus the virus could potentially escape the low-level immunity in older individuals and lead to more effective virus replication and severe disease outcomes.

COVID-19 mRNA vaccination has been actively implemented globally. It has been effective in inducing serum antibody responses and protect against SARS-CoV-2 infection in clinical and animal studies [35,36,37]. In our aged mice model, their serum-neutralizing antibody titre was significantly lower than that of the vaccinated young mice, with 2 of 6 aged mice having no detectable neutralizing antibody titre after two-dose vaccination. Our data demonstrated that even the highly immunogenic mRNA vaccine was unable to induce satisfactory antibody responses in aged mice. Subsequently, abundant viral antigen expression and infectious virus titre accompanied by severe tissue destruction were found in the NT of aged mice after the virus challenge. Moreover, the finding of the lower frequency of IgG-secreting cells and IFN-γ-secreting cells in the spleen of vaccinated aged mice at 2 days post virus challenge aligns with the findings observed from re-infected aged mice. They suggest a poor immune memory recall upon virus challenges [38]. Vaccine breakthrough infections of SARS-CoV-2 have been reported among healthy persons and were generally mild, which did not require hospitalization [39]. Our results could imply that, vaccine breakthrough infection could occur more frequently in older individuals than the younger population. Together with the suboptimal immune memory recall, the elderly had a higher possibility of developing severe diseases. While comparing the IgG-producing cell and IFN-γ-producing cell responses in the spleen of aged mice immunized by the primary infection with vaccination, we assume that though both B and T cell immune responses are impaired in aged mice compared to young mice. mRNA vaccination conferred better T cell responses than natural virus infection in aged mice. This is in line with the findings that better vaccine-induced protection in the elderly population is correlated with more Th1 T-cell responses [40]; this again supports the importance of vaccination in the protection of the elderly population against SARS-CoV-2.

Overall, we demonstrated that ageing resulted in weaker protective immune response or recall, more SARS-CoV-2 pathology, higher risk of re-infection, and a higher chance of vaccine breakthrough infections. Our study suggests that tailored treatment and prevention strategies for the advanced aged population should be investigated and implemented [41,42]. Approaches to optimize the effectiveness of vaccination in older individuals, potentially by adding more booster doses and/or with other adjuvants, warrant further investigation.
